# Understanding the life experience of Barth syndrome from the perspective of adults: a qualitative one-on-one interview study

**DOI:** 10.1186/s13023-019-1200-8

**Published:** 2019-11-07

**Authors:** Iyar Mazar, Jonathan Stokes, Sarah Ollis, Emily Love, Ashlee Espensen, Peter G. Barth, John H. Powers, Alan L. Shields

**Affiliations:** 1 Employed at Adelphi Values at the time of the conduct of the research, Boston, MA USA; 2 Adelphi Values, 290 Congress St., 6th floor, Boston, MA USA; 3 Department of Pediatric Neurology, Emma Children’s Hospital, University of Amsterdam, Amsterdam, Netherlands; 4 George Washington University School of Medicine, Washington DC, USA

**Keywords:** Barth syndrome, Qualitative research, Health outcomes, Quality of life, Natural history, X-linked disease, Rare disease, Aging

## Abstract

**Background:**

Barth syndrome (BTHS, OMIM 302060) is a rare, life-threatening, x-linked genetic disorder that occurs almost exclusively in males and is characterized by cardiomyopathy, neutropenia, skeletal muscle myopathy primarily affecting larger muscles, and shorter stature in youth. A greater number of individuals with BTHS are now surviving into adulthood due to advancements in diagnosis and disease management. Given these improvements in life expectancy, understanding the disease experience over time has become increasingly important to individuals with the condition, treatment developers, and regulatory agencies. A study was conducted to explore the experience of BTHS from the perspective of adult males at least 35 years of age with the condition via in-depth qualitative interviews.

**Results:**

Findings showed that adults with BTHS experienced a variety of signs/symptoms with variable onset and severity throughout their lives, the most frequently reported being the symptoms of tiredness, muscle weakness, and a fast and/or irregular heart rate, and the sign of short stature in youth. These signs/symptoms negatively impacted individuals’ emotional, physical, social, and role functioning. Tiredness and weakness impacted some individuals’ physical functioning from an early age and into adulthood. These symptoms generally worsened over time, increasingly interfering with individuals’ ability to fully participate in paid and unpaid labor and to partake in family and leisure activities.

**Conclusions:**

This research complements recent studies characterizing the potentially degenerative and progressive nature of BTHS and can encourage future research into the natural history and progression of BTHS in untreated individuals. Participants’ interview responses revealed a range of symptoms and the potential for multiple impacts on individuals’ physical, social, emotional, and role functioning as a result of BTHS symptoms, yet also revealed variability in severity of experience as well as the possibility of resilience and adaptation to the condition.

## Background

Barth syndrome (BTHS, OMIM 302060) is a rare, X-linked recessive disease, therefore occurring almost exclusively in males. BTHS is caused by mutations in the tafazzin (TAZ) gene with the unique biochemical signature of decreased levels of mature cardiolipin and accumulation of monolysocardiolipin. This is caused by deficiency of an enzyme encoded by tafazzin affecting the structure and function of the inner mitochondrial membrane [1–3]. Abnormalities in mitochondria can cause cardiomyopathy, neutropenia, skeletal myopathy, fatigue, short stature, exercise intolerance, and feeding problems [1–4]. While prevalence of the disease is not well documented [3], it is estimated that the condition affects approximately one in every 300,000 to 400,000 individuals globally [1, 2], with symptoms varying in presentation and severity. BTHS can be fatal in childhood due to heart failure or uncontrollable infection, and individuals with BTHS who survive to adulthood experience a shortened life expectancy [3–5].

Due to the early onset of symptoms and shortened life expectancy associated with the condition, research in BTHS is most often focused on the experiences of younger individuals (i.e., children and adolescents) [6, 7], which can result in the misperception that BTHS affects only a pediatric population. Further complicating our understanding of the course of BTHS are the often-observed improvements in general health and the steadying of cardiomyopathy and related heart issues following disease involvement. This “honeymoon phase” typically occurs in middle childhood or adolescence and is problematic for a variety of reasons, including that it further contributes to missed chances for appropriate diagnosis and treatment and for a greater understanding of disease course [8].

In recent decades, however, greater numbers of individuals with BTHS are surviving into adulthood, mainly due to improvements in diagnosis and disease management [5, 8]. As a result of improved life expectancy, a new age group of middle-aged adults (referred to hereafter as adults) with BTHS has emerged, which requires professional attention. Therefore, understanding the experience of BTHS over time is increasingly important to individuals with the condition, treatment developers, and regulatory agencies. It is the purpose of this research to draw attention to this age group. Until recently, very little information has been available regarding the experiences of adults with BTHS and hence, understanding of disease progression is limited [9]. Thus, the objective of this study was to explore the lived experience of BTHS from the perspective of adult men over the age of 35 with the disease. These results can inform understanding of disease progression and symptom trajectory over time and, ultimately, help to define the important and relevant health outcomes and needs of adults with BTHS.

## Methods

This qualitative interview study was approved by an independent review board on 24 April 2018 (WIRB Study Number 1133398). The research team collaborated with the Barth Syndrome Foundation (BSF) to recruit a convenience sample of participants. The BSF contacted individuals with BTHS who were 35 years of age or older in April 2018 and who had not yet been provided the opportunity to share their subjective disease-related experiences as part of a previously conducted qualitative research study. The BSF knew of 12 individuals with BTHS over the age of 35 at the time of this study, three of whom the BSF had previously lost contact with and two who had participated in the prior qualitative study. This resulted in a total of seven individuals who were contacted to participate in the study and all agreed to partake. In the next five years, the BSF estimates that there may be 19 total individuals 35 years of age and older with BTHS.

Each individual provided informed consent prior to participation. Individuals were able to invite others to join their interview if they chose (e.g., family members, translators). All but one of the individuals who participated in the interviews were members of the Barth Syndrome Foundation registry, which includes documentation of a genetically confirmed diagnosis of BTHS consisting of mutation analysis of the TAZ gene.

Interviews were conducted by trained researchers in a semi-structured fashion, allowing for the elicitation of spontaneous information regarding the experience of BTHS symptoms and their progression over time. BTHS disease-related impacts to daily living and overall quality of life, as well as coping strategies for managing impacts, were also explored. Each interview was audio-recorded, transcribed, anonymized, and analyzed in ATLAS.ti, a qualitative data analysis program. Analysis was conducted in two parts. First, each participant’s responses relevant to the research questions discussed during the interview were coded. Second, these coded responses were aggregated and evaluated across the total sample. For the first stage of analysis, three of the four members of the research team who conducted the interviews independently coded each transcript. Coding consisted of identifying themes pertinent to the research questions and tagging, or “coding,” the relevant participant quotes in each transcript. The research team met to harmonize the codes (i.e., review and consolidate any similar codes) in order to determine preliminary themes for further analysis. All coded text was aggregated across the sample and evaluated by the research team. All the coded responses were reviewed by theme (e.g., emotional impacts) in order to arrive at final categorizations and characterizations of participants’ experiences with BTHS. The data were evaluated to determine (1) frequency of reported concepts (e.g., how many participants reported experiencing a given symptom or impact) and (2) the content and meaning of participants’ responses. Preliminary findings were presented to the BSF community and shared with the study participants in the form of a conference poster developed for the BSF’s 2018 International Scientific, Medical & Family Conference [10].

## Results

In total, seven 60-min telephone interviews were conducted with individuals (ages 35 years and older) with BTHS living in Europe (*n* = 5) and the United States (*n* = 2) between May and June 2018. Participants’ ages ranged from 37.2 to 58.6 years with a mean of 51.3 years of age. Notably, the majority of participants in this study were 50 years of age or older (*n* = 5, 71.4%). Most individuals (n = 5, 71.4%) reported currently living with family or friends and the same number reported their highest level of educational attainment being a high school diploma (GED) or less. Over half (*n* = 4, 57.1%) were employed either full- or part-time, while over a third were either on disability leave or unemployed (*n* = 3, 42.9%). While demographic and health questions regarding individuals’ marital/parental status, current and/or prior medication use, and family history with BTHS were not directly asked as part of the interviews, this information was spontaneously reported by most participants. Six participants (85.7%) discussed having currently or previously lived in marital relationships, and five of these participants (71.4%) reported being a parent to at least one biological child (two participants did not spontaneously discuss having children). Six participants (85.7%) discussed currently taking a variety of cardiac-related medications and none reported having undergone a heart transplant. Six participants (85.7%) spontaneously reported having other affected family members with BTHS (e.g., siblings, nephews). See Table 1 for participant self-reported demographic and health information.

**Table 1:** Participant self-reported demographic and health information

Characteristic	Total (N = 7) n/N^a^
Current age/Age at diagnosis	
Mean (SD)	51.3 (7.1)/42.4 (8.3)
Range	37.2–58.6/33.0–54.0
Age at onset of Barth Syndrome symptoms	
Under 6 years old	5/7
18 years or older	1/7
Unknown	1/7
Living situation	
Living with family or friends	5/7
Living alone	1/7
Living with a caregiver or in a caregiving facility	1/7
Work status^b;c^	
Working full-time	2/7
Working part-time	2/7
On disability	3/7
Unemployed	1/7
Other (Volunteer)	1/7
Education level	
High school diploma (or GED) or less	5/7
College or university degree	1/7
Post-graduate degree	1/7

^a^Unless otherwise noted

^b^Counts not mutually exclusive

^c^Occupations reported by participants included office work, food service, volunteer work, and manual labor

### Diagnosis and symptom experience

Disease-related symptoms are defined as experiences that can be best assessed by and/or noticed and known only by the patient [11]. Most individuals in this study (*n* = 5, 71.4%) reported experiencing symptom onset before six years of age, and all but one reported experiencing some level of BTHS symptoms in childhood, ranging from mild symptoms with little impact to severely limiting symptoms. All individuals were diagnosed in mid-adulthood; for those with severe, early-onset symptoms, diagnosis explained previously unanswered questions regarding their health and physical limitations, information that would have been useful earlier in life (e.g., to help explain one’s limitations to others). For those with minimal and/or late onset BTHS, diagnosis was unexpected. Some participants (*n* = 3, 42.9%) reported that an earlier diagnosis may have informed important life decisions with respect to career, education, and family planning. Table 2 presents each participant’s self-reported BTHS sign/symptom and impact experiences. Excerpts reflecting select participant descriptions of their BTHS sign and symptom experiences are provided in Table 3 in the Appendix.

**Table 2:** Participants’ self-reported BTHS sign/symptom and impact experiences

Participant and current work status	Age at symptom onset	Initial signs/symptoms	Current signs/symptoms	Impact domains reported
Participant 01 Unemployed and disability	Under 6 years old	• Cardiovascular (enlarged heart) • Tiredness • Muscle weakness	• Cardiovascular (fast heart rate) • Tiredness • Muscle weakness • Shortness of breath	• Emotional • Work/school • Physical • Family • Social • Role function • Financial • Activities of daily living • Leisure
Participant 02 Part-time employment	Unknown	• Cardiovascular (fast heart rate)	• No current symptoms reported	• Emotional • Work/school • Family
Participant 03 Full-time employment	Under 6 years old	• Cardiovascular (described as fluid in heart, likely pericardial effusion) • Tiredness	• No current symptoms reported	• Emotional • Family
Participant 04 Part-time employment and volunteer work	Under 6 years old	• Tiredness • Muscle weakness • Short stature • Impaired immune system • Overweight • Difficulty eating • Difficulty concentrating	• Tiredness • Muscle weakness • Impaired immune system • Difficulty eating	• Emotional • Work/school • Physical • Social • Role function • Activities of daily living
Participant 05 Disability	Under 6 years old	• Tiredness • Muscle weakness • Short stature • Sensitivity to temperature • Enlarged cheeks	• Cardiovascular • Tiredness • Muscle weakness • Muscle pain • Sensitivity to temperature	• Emotional • Work/school • Physical
Participant 06 Disability	18 years or older	• Tiredness • Muscle weakness • Short stature • Difficulty eating	• Cardiovascular (fast heart rate, fluid around heart, likely pericardial effusion) • Tiredness • Muscle weakness • Short stature • Difficulty eating	• Emotional • Work/school • Physical • Family • Social • Role function • Leisure
Participant 07 Working full-time	Under 6 years old	• Tiredness • Muscle weakness • Short stature • Impaired immune system • Overweight • Impaired gait	• Cardiovascular (irregular heart rate) • Tiredness • Muscle weakness • Muscle pain • Dizziness • Impaired gait	• Emotional • Work/school • Physical • Social • Financial

**Fig. 1:**
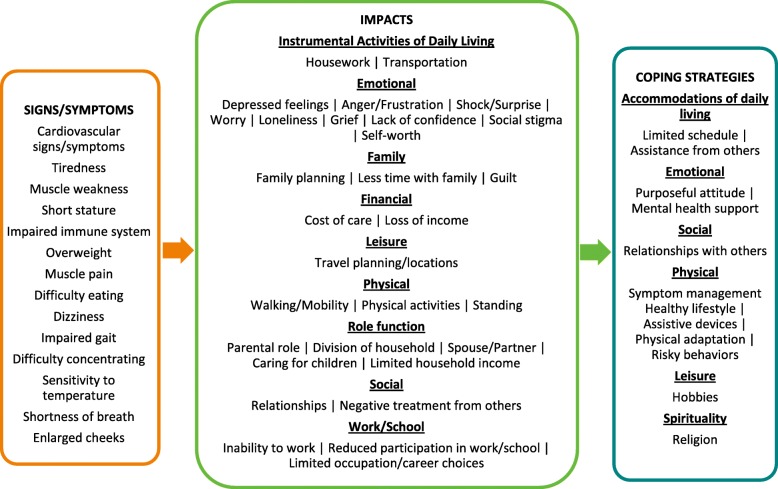
BTHS symptoms, impacts, and coping strategies in adults over 35 years of age

When asked to discuss the signs/symptoms that they experienced throughout their lives, participants reported a total of 14 unique signs/symptoms, all of which are listed in the conceptual model in Fig. 1. Of those 14 total signs/symptoms, participants most frequently reported currently experiencing the symptoms of tiredness (e.g., low energy, sleepiness, fatigue; *n* = 5, 71.4%), muscle weakness (e.g., difficulty lifting/carrying objects, lack of strength to complete physical activities; n = 5, 71.4%), and fast and/or irregular heart rate (*n* = 4, 57.1%). The sign of short stature (n = 4, 57.1%) was frequently reported by participants as being experienced primarily in youth, and cardiovascular issues (e.g., enlarged heart) were frequently reported (*n* = 6, 85.7%) as being relevant throughout the life course. Two of the seven participants reported experiencing no current signs or symptoms. The severity of cardiovascular signs/symptoms and issues was variable between and within participants (e.g., ranging from fast/irregular heart rate to heart failure), and short stature improved in late adolescence. Tiredness and muscle weakness were experienced throughout the life course, posed functional limitations in youth and into adulthood, and generally worsened over time.

### Impacts to quality of life

Disease-related impacts are defined as the patient-centered effects, burdens, and/or limitations that acute and chronic signs and symptoms have on individuals’ daily lives [12]. A total of 29 impacts across nine domains were elicited from individuals with BTHS in this study, with the most frequently reported impact domains being emotional (*n* = 7, 100%), work/school (*n* = 6, 85.7%), and physical (*n* = 5, 71.4%). The most frequently reported impacts were reduced participation in work/school (n = 6, 85.7%), walking/mobility limitations (n = 5, 71.4%), inability to work (n = 5, 71.4%), and impacted physical activities (*n* = 4, 57.1%). Three participants (42.9%) each reported experiencing anger/frustration, depressed feelings, limited occupation/career choices, negative treatment from others, limitations to parental role (e.g., difficulty providing care for one’s children and/or partaking in recreational activities with children), and shock/surprise at diagnosis. Most impacts were relevant to individuals throughout the life course, with those pertaining to work (i.e., unpaid and paid labor) being most relevant to individuals’ lives currently. Tiredness and weakness impacted individuals’ physical functioning, often on a daily basis, as well as their ability to partake in paid/unpaid labor, and were associated with negative social interactions. Excerpts reflecting select participant descriptions of their impact experiences related to BTHS are provided in Table 4 in the Appendix.

### Coping strategies

Coping strategies are defined as the emotional, cognitive, and/or behavioral means by which individuals manage the symptoms and impacts of living with a condition [13]. A total of 12 coping strategies across six domains were reported by adults with BTHS in this study, with the most frequently reported domains being physical (*n* = 7, 100.0%), emotional (*n* = 6, 85.7%), and accommodations of daily living (n = 6, 85.7%). The most frequently reported specific coping strategies were maintaining a purposeful attitude (n = 6, 85.7%), symptom management (n = 6, 85.7%), limiting one’s schedule (*n* = 5, 71.4%), and pursuing hobbies (*n* = 4, 57.1%). One individual reported previously relying on negative coping strategies (e.g., substance abuse and engaging in risky health behaviors). The use of assistive devices to manage difficulties with mobility and to complete daily tasks was reported by three participants (42.9%) who discussed utilizing aids such as an electric bicycle, a motorized scooter, a cane, and a pulley system for lifting heavy objects at work (*n* = 1, 14.3% each). Positive impacts associated with BTHS were also reported by participants; the most frequently reported was developing and maintaining strong relational bonds with family/community members as a result of BTHS (*n* = 4, 57.1%). Excerpts reflecting select participant approaches to coping with BTHS are provided in Table 5 in the Appendix.

## Discussion

Adults with BTHS experienced multiple symptoms with variable onset and severity throughout the life course; these symptoms negatively impacted individuals’ emotional, physical, social, and role functioning (see Fig. 1). Individuals’ coping strategies included adapting their daily routines, managing their physical limitations and emotional responses, and relying on social support and personally fulfilling hobbies. Some individuals reported that they currently experienced minimal impacts to daily life or that they experienced issues only temporarily at symptom onset, whereas other participants reported that their symptom progression had resulted in limitations that had become increasingly more significant in terms of physical functioning and independent living (e.g., reliance on others for income, household chores). The varying degree of impact that participants reported was rooted in the same set of symptoms (e.g., tiredness, weakness); however, the level of impact was dependent upon the extent of symptom severity. Those individuals who were not currently symptomatic experienced impacts in fewer domains, primarily relating to shock around diagnosis that impacted family life and emotional functioning, as well as impacts to work due to prior cardiovascular issues. Thus, for measurement purposes (e.g., assessing treatment benefit, disease progression over time), the same set of symptom and impact concepts are relevant to evaluate in this target patient population (i.e., adults with BTHS).

Tiredness and weakness specifically impacted some individuals’ physical functioning from an early age. These symptoms generally worsened over time, increasingly interfering with individuals’ ability to fully participate in paid and unpaid labor and to partake in family and leisure activities. Despite being diagnosed late in life, individuals found means for coping with the impacts of their condition and were able to attain meaningful goals such as making contributions to work and family.

There are several important limitations inherent to the present research design that must be acknowledged when interpreting these results, and which may serve to inform future studies seeking to build upon this work. One consideration when reviewing these findings is that only those individuals who survived into adulthood were included in this sample; thus, their disease trajectories and experiences may or may not reflect those of individuals who did not survive to 35 years of age. On the other hand, there also exists the potential that individuals with less severe, undiagnosed cases of BTHS were not included in this study.

This study relied exclusively on self-reported health data, which was deemed an appropriate methodology for addressing the objective of understanding the experiences of adults with BTHS that are best known to the participants (e.g., how symptoms feel and change over time). However, the inclusion of clinical information (e.g., from a review of medical records) could have further contextualized these qualitative data, and future studies may analyze individuals’ self-reported experiences in relation to the clinical severity of their condition. For instance, Bowron et al. (2015) identified unique disease profiles in select individuals whose severity of BTHS was relatively low compared to other individuals with the condition [14]. Of the seven individuals with these unique profiles, five had excellent tolerance to exercise and two were asymptomatic. Therefore, this kind of clinical information, coupled with participants’ reports on their perceived health experiences, could be analyzed in order to better understand whether the present findings accurately reflect how BTHS develops and potentially progresses over time for those who survive to age 35 and older. For example, self-reported data in conjunction with information on participants’ clinical characteristics could be used in future research to develop case studies for BTHS across the spectrum of known disease profiles. These case studies could capture challenges in individuals’ diagnostic journeys and their overall disease trajectory.

Another potential limitation of this study is its sample size, which may raise concerns regarding the utility of the findings. Nevertheless, qualitative research designs are inherently small n studies and researchers have both suggested and demonstrated that these kinds of studies and sample sizes can produce trustworthy and valuable results [15, 16] with respect to broadening understanding of health-related experiences. Moreover, and as noted previously, this sample of seven interviewed participants reflects almost all of the known individuals with BTHS over age 35, indicating that the results can be considered representative of men diagnosed with BTHS who have survived to this age to date.

Finally, due to the few known individuals with BTHS in this adult age group, a small sample size was anticipated for this study, and therefore no a priori statistical tests were specified. Quantitative evaluations in this study were limited to the calculation of descriptive statistics to characterize the study sample and to the presentation of frequencies and percentages in-text for demographic and health information as well for the sign, symptom, impact, and coping concepts reported by participants.

Despite these limitations, this study serves as an attempt to begin understanding the trajectory of BTHS over the life course. Given the rarity of this condition, the seven participants’ interview responses begin to shed light on the within- and between-person variability of the condition over time, and provide valuable directions for future research that can build upon this present work in BTHS.

### Clinician perspective

The findings noted in this study can be useful for both clinical practice and for the design of clinical trials for treatments in BTHS.

For clinical practice, the data presented here can provide helpful information for clinicians to query patients already diagnosed with BTHS about specific symptoms related to BTHS, and guide treatment of the disease or its complications. An assessment of the impacts of the disease on patients’ lives can aid clinicians in helping patients access social supports. The data also show that some patients were diagnosed later in the course of their disease, and findings of such symptoms, along with low circulating neutrophils in some cases, may be a clue for clinicians to include BTHS in the differential diagnosis of these symptoms and to initiate diagnostic testing such as an assay measuring cardiolipin ratio [8].

For clinical research, the findings in this study can be used in observational studies to evaluate the clinical course of the illness. This study also provides initial evidence on the concepts that should be included in a draft patient-reported outcome (PRO) instrument to evaluate symptom and impacts of BTHS. As noted, the patient population included in this study may represent a subset of patients with the disease, but the evidence presented here is valid in relation to context of use of the types of patients included in this study. This study could form the basis for the content validity of a PRO instrument for evaluation of treatment effects in medical interventions in this patient subset.

## Conclusions

The purpose of this research was to explore and document the experiences of adults with BTHS and to raise awareness among various stakeholders (e.g., clinicians, researchers) regarding BTHS. This research complements recent studies characterizing the potentially degenerative and progressive nature of the condition [17] and can encourage future research into the natural history and progression of BTHS in untreated individuals in order to address additional questions (e.g., researchers may attempt to determine whether a second “honeymoon” period exists in BTHS in middle age). Additionally, results presented here should encourage researchers to better understand BTHS among individuals older than 35 and, further, incorporate the experiences of different types of individuals currently approaching the age of 35 who were not included in this study. Participants’ interview responses revealed a range of symptoms and the potential for multiple impacts on individuals’ physical, social, emotional, and role functioning as a result of BTHS symptoms, yet also revealed variability in severity of experience as well as the possibility of resilience and adaptation to the condition.

## Data Availability

All data generated or analyzed during this study are included in this published article.

## Appendix

**Table 3:** Sample quotations for BTHS signs/symptoms and disease progression

Fast heart rate	
“I have a very fast heart and some periods of feeling that I have very little breath.…Sometimes when I was lying in bed and I thought my heart was going really fast, but you didn’t think something special about it, at least at that point …”	
Tiredness	
“You have some energy for a period but then it goes very fast…. All days are the same for me. I have energy for a part of the day. And when the mid-day comes I feel the energy is fading away …”	
Muscle weakness	
“Muscle weakness and pain, like I said, especially with the running and football it was the wind sprints; I usually couldn’t finish the wind sprints with the other guys. I’d go as far as I could, but I couldn’t do as much as they could. And I knew that for whatever reason that I got tired quicker and that it hurt me more, but I didn’t know why.”	
Short stature	
“Just everything went slower…. In every way you were slower in developing your body, especially when you become [a] teen.… I just had a kid body.… I think when at 24 yes, it was, yeah, still I’m small but you can see now I have a more male body than back then.”	
Symptom progression	
“Well I think it becomes a bit worse in my muscle strength. It goes very slowly out, but I think I’ve seen all the years by, I think it’s getting worse.” “I started getting weaker when I was around 50. And it’s gotten a lot worse in the last couple of years. These days, if I do two or three hours of physical labor, it’s going to take me two or three days to recover …”	

**Table 4:** Sample quotations for disease-related impacts

Emotional	
“Sometimes I think I have depression, but is that related to Barth syndrome? I don’t know. It can also be related to other issues.… it’s too big to say [it] destroyed your youth, but, yeah, it’s hard, it’s painful.”	
Work/school	
“… really feeling tired and not having the strength to follow others.… I didn’t have to participate in sports in school either because of that.… Barth syndrome was the cause that I started working part-time. Because I noticed I couldn’t keep up with things.... Otherwise I would have worked five days a week like anyone else … that changed a bit over the years because the limitations got a bit more over time.”	
Physical	
“We noticed that something was wrong because when we were going on holidays, we never went to areas … with mountains or hills because... When I have to walk up that, that’s really a problem. And that was already when I was very young … so I walk shorter than I used to. I cycle shorter than I used to. Walking stairs is more difficult. And then the last years, problems with the heart … other people do a lot of things for me, if they cause a strain. I knew that physical tasks, exercise, and all of that stuff, I knew it was a lot harder for me than it was for the other kids. Didn’t know why …”	
Social	
“I was teased a lot by the other children, bullied, and all that.… They couldn’t even say, well he’s like this because he has this condition, you know? There, there wasn’t no excuse for it. I was just different …”	
Role function	
“I play with the kids … but it takes a lot more. They need a lot more attention and a lot more care. Because I work in the morning. I do a little bit of household in the mid-day. And then I have no energy left … the kids come from school and they also want to have some attention. That has a major impact here at home …”	

**Table 5:** Sample quotations for coping strategies

Accommodations of daily living	
“Everything at a slow pace. In short, I take my time…. and when it comes to [something] for which you need strength, I have to mostly leave it to someone else. And I just need help on other things…. It’s really the resting afterwards and the slow pace when I am doing those things. And I plan my time so I can do things at a slow pace.”	
Emotional	
“Life gets better.… I mean I don’t know if life gets better, but at least you get better able to deal with it.… Don’t give up. It gets better.”	
Social	
“Our therapy was coming together a lot and we talked a lot together as a family.… So the power of our family, we are very open to each other. We communicate about everything with all this and that’s our strength now …”	
Physical	
“Well for this bad heart, he really pays attention to his lifestyle and the food he takes. And he tries to walk regularly.”	
Leisure	
“The positive point of Barth syndrome is that you are a bit lonely so you do things at home … I can play my own emotion into music …”	

## Abbreviations

BTHS: Barth syndrome
PRO: Patient-reported outcome

## References

[1] Genetics Home Reference. Barth syndrome 06/14/2016 [updated 06/14/2016]. Available from: https://ghr.nlm.nih.gov/condition/barth-syndrome.

[2] Barth Syndrome Foundation. Overview of Barth Syndrome 6/14/2016 [updated 6/14/2016]. Available from: https://www.barthsyndrome.org/about-barth-syndrome.

[3] Spencer CT, Bryant RM, Day J, Gonzalez IL, Colan SD, Thompson WR (2006). Cardiac and clinical phenotype in Barth syndrome. Pediatrics..

[4] Bione S, D'Adamo P, Maestrini E, Gedeon AK, Bolhuis PA, Toniolo D (1996). A novel X-linked gene, G4.5. Is responsible for Barth syndrome. Nat Genet.

[5] Jefferies JL (2013). Barth syndrome. Am J Med Genet C: Semin Med Genet.

[6] Rigaud C, Lebre AS, Touraine R, Beaupain B, Ottolenghi C, Chabli A (2013). Natural history of Barth syndrome: a national cohort study of 22 patients. Orphanet journal of rare diseases.

[7] Roberts AE, Nixon C, Steward CG, Gauvreau K, Maisenbacher M, Fletcher M (2012). The Barth syndrome registry: distinguishing disease characteristics and growth data from a longitudinal study. Am J Med Genet A.

[8] Clarke SL, Bowron A, Gonzalez IL, Groves SJ, Newbury-Ecob R, Clayton N (2013). Barth syndrome. Orphanet journal of rare diseases..

[9] Ronvelia D, Greenwood J, Platt J, Hakim S, Zaragoza MV (2012). Intrafamilial variability for novel TAZ gene mutation: Barth syndrome with dilated cardiomyopathy and heart failure in an infant and left ventricular noncompaction in his great-uncle. Mol Genet Metab.

[10] Stokes J, Mazar I, Ollis S, Love E, Espensen A, Shields AL. Understanding the life experience of Barth syndrome from the perspective of older individuals. Barth Symptom Foundation international scientific, Medical & Family Conference; Clearwater, FL. July 2018:16–20.

[11] US Department of Health and Human Services, Food and Drug Administration, Center for Drug Evaluation and Research, Center for Biologics Evaluation and Research, Center for Devices and Radiological Health. Guidance for industry patient-reported outcome measures: use in medical product development to support labeling claims. 2009.

[12] Wilson IB, Cleary PD (1995). Linking clinical variables with health-related quality of life. A conceptual model of patient outcomes. JAMA..

[13] Folkman S, Lazarus RS, Dunkel-Schetter C, DeLongis A, Gruen RJ (1986). Dynamics of a stressful encounter: cognitive appraisal, coping, and encounter outcomes. J Pers Soc Psychol.

[14] Bowron A, Honeychurch J, Williams M, Tsai-Goodman B, Clayton N, Jones L (2015). Barth syndrome without tetralinoleoyl cardiolipin deficiency: a possible ameliorated phenotype. J Inherit Metab Dis.

[15] Patrick DL, Burke LB, Gwaltney CJ, Leidy NK, Martin ML, Molsen E (2011). Content validity-establishing and reporting the evidence in newly developed patient-reported outcomes (PRO) instruments for medical product evaluation: ISPOR PRO good research practices task force report: part 1-eliciting concepts for a new PRO instrument. Value Health.

[16] Turner-Bowker DM, Lamoureux RE, Stokes J, Litcher-Kelly L, Galipeau N, Yaworsky A (2018). Informing a priori sample size estimation in qualitative concept elicitation interview studies for clinical outcome assessment instrument development. Value Health.

[17] Thompson WR, DeCroes B, McClellan R, Rubens J, Vaz FM, Kristaponis K (2016). New targets for monitoring and therapy in Barth syndrome. Genetics in medicine : official journal of the American College of Medical Genetics.

